# *Nigella sativa* L. and COVID-19: A Glance at The Anti-COVID-19 Chemical Constituents, Clinical Trials, Inventions, and Patent Literature

**DOI:** 10.3390/molecules27092750

**Published:** 2022-04-25

**Authors:** Mohd Imran, Shah Alam Khan, Abida, Mohammed Kanan Alshammari, Saif M. Alkhaldi, Fayez Nafea Alshammari, Mehnaz Kamal, Ozair Alam, Syed Mohammed Basheeruddin Asdaq, A. Khuzaim Alzahrani, Shahamah Jomah

**Affiliations:** 1 Department of Pharmaceutical Chemistry, Faculty of Pharmacy, Northern Border University, Rafha 91911, Saudi Arabia; aqua_abkhan@yahoo.com; 2 Department of Pharmaceutical Chemistry, College of Pharmacy, National University of Science and Technology, Muscat 130, Oman; shahalam@nu.edu.om; 3 Department of Pharmaceutical Care, Rafha Central Hospital, Rafha 91911, Saudi Arabia; ii_kanan101@outlook.com; 4 Department of Pharmaceutical Care, King Khalid Hospital in Majmaah, Riyadh 76312, Saudi Arabia; ph.saif.alkhaldi@gmail.com; 5 Community Pharmacist, Al-Dawaa Pharmacies, Hafer Albatin 39911, Saudi Arabia; fayezalshammari96@gmail.com; 6 Department of Pharmaceutical Chemistry, College of Pharmacy, Prince Sattam Bin Abdulaziz University, Al-Kharj 11942, Saudi Arabia; mailtomehnaz@gmail.com; 7 Medicinal Chemistry and Molecular Modelling Lab, Department of Pharmaceutical Chemistry, School of Pharmaceutical Education and Research, Jamia Hamdard, New Delhi 110062, India; oalam@jamiahamdard.ac.in; 8 Department of Pharmacy Practice, College of Pharmacy, AlMaarefa University, Dariyah, Riyadh 13713, Saudi Arabia; 9 Department of Medical Laboratory Technology, Faculty of Applied Medical Sciences, Northern Border University, Arar 91431, Saudi Arabia; akaalz@nbu.edu.sa; 10 Pharmacy Department, Sulaiman Al-Habib Medical Group, Riyadh 11643, Saudi Arabia

**Keywords:** *Nigella sativa* L., black seed, SARS-CoV-2, COVID-19, clinical trial, invention, patent

## Abstract

COVID-19 has had an impact on human quality of life and economics. Scientists have been identifying remedies for its prevention and treatment from all possible sources, including plants. *Nigella sativa* L. (NS) is an important medicinal plant of Islamic value. This review highlights the anti-COVID-19 potential, clinical trials, inventions, and patent literature related to NS and its major chemical constituents, like thymoquinone. The literature was collected from different databases, including Pubmed, Espacenet, and Patentscope. The literature supports the efficacy of NS, NS oil (NSO), and its chemical constituents against COVID-19. The clinical data imply that NS and NSO can prevent and treat COVID-19 patients with a faster recovery rate. Several inventions comprising NS and NSO have been claimed in patent applications to prevent/treat COVID-19. The patent literature cites NS as an immunomodulator, antioxidant, anti-inflammatory, a source of anti-SARS-CoV-2 compounds, and a plant having protective effects on the lungs. The available facts indicate that NS, NSO, and its various compositions have all the attributes to be used as a promising remedy to prevent, manage, and treat COVID-19 among high-risk people as well as for the therapy of COVID-19 patients of all age groups as a monotherapy or a combination therapy. Many compositions of NS in combination with countless medicinal herbs and medicines are still unexplored. Accordingly, the authors foresee a bright scope in developing NS-based anti-COVID-19 composition for clinical use in the future.

## 1. Introduction

COVID-19, a pandemic transmissible sickness, is triggered by SARS-CoV-2. As of 1 April 2022, this disease has infected >488 million individuals, led to about 6.16 million mortalities, and affected the quality of life and worldwide economy since its inception in 2019 [1]. COVID-19 cases have been documented around the globe, wherein the United States, India, Brazil, France, and Germany have reported the majority of cases [1]. The clinical features of COVID-19 are similar to normal flu (cough, sore throat, high fever, headache, sneezing, myalgia, breathlessness, etc.), and it mainly targets the respiratory system, leading to acute respiratory distress syndrome. If untreated on time, COVID-19 may be fatal, and the patient may die due to pneumonia, respiratory failure, and damage to other vital organs due to cytokine storm [2]. Some vaccines (Janssen, Moderna, and Pfizer-BioNTech), new drugs (remdesivir, molnupiravir, favipiravir, and a combination of nirmatrelvir with ritonavir), and supportive therapy (a combination of paracetamol, vitamin C, and zinc) have been established to prevent/treat COVID19 [2,3,4]. The drugs used to prevent/treat COVID-19 act on different enzymes/proteins of SARS-CoV-2, including RNA-dependent-RNA-polymerase, helicase, main protease, non-structural proteins (Nsp9 and Nsp15), and RNA replicase [2,3,4]. Many new drugs and their combinations are also in clinical trials [3,4]. Despite the development of many vaccines and some drugs, the battle against COVID-19 is far from over. The identification and the spread of new contagious SARS-CoV-2 strains (α, β, γ, δ, Omicron, etc.) also pose challenges to the existing vaccines and treatment of COVID-19 [5]. Accordingly, the development of alternative and supporting therapies has been perceived as a promising way to combat COVID-19 [6,7,8,9,10]. Many studies have demonstrated the effectiveness of health supplements (zinc, vitamin C, vitamin D, etc.) and herbal products (black seed, honey, ginger, garlic, etc.) to prevent/treat COVID-19 [11,12]. This review highlights the anti-COVID-19 potential of *Nigella sativa* L. (NS) and its major chemical constituents.

## 2. Materials and Methods

The non-patent literature search for the titled review was performed on the PubMed database and Sci-finder database, utilizing keywords COVID-19 or SARS-CoV-2 in combination with different keywords related to *N. sativa* (Black cumin, Black seed, and Kalonji). The relevant non-patent literature citing the prophylactic/therapeutic use of *N. sativa* against COVID-19 was reviewed and cited in this review. The patent searching was performed utilizing different patent databases (Sci-Finder, Espacenet, Patentscope, and USPTO). The patents/patent applications were segregated according to their patent family, and duplicate patent families were removed. The patent applications that exclusively provide clear information about the use of *N. sativa* or *N. sativa*-based inventions to treat COVID-19 have been included in this review. The patent searching results are provided as Supplementary Material (Table S1).

## 3. *N. sativa* L.

### 3.1. Introduction

Phytotherapy is one of the alternative approaches explored by the scientific community to discover and develop preventive and/or therapeutic regimens against the menace of SAR-CoV-2. Amongst thousands of ethnomedicinal plants which are known to be effective against infectious diseases and alleviate acute respiratory symptoms, *N. sativa* and its phytoconstituents are one of the extensively studied natural substances.

*N. sativa* belonging to the family Ranunculaceae is a nutrient-rich, annual flowering medicinal plant of significant historical and religious importance. It is usually recognized as black seed or black cumin in English, Habbatul barakah in Arabic, and Kalonji in Hindi and Urdu [13]. Its nutritional value and a wide array of therapeutic properties have been known to mankind since ancient times. Muslims consider black seed as a panacea, i.e., a remedy that can cure all diseases except death, while traditional medicine practitioners believed it to be ‘the herb from heaven’ [14]. Its beneficial uses are also mentioned by the 10th-century physician Avicenna in his book ‘The Canon of medicine’ [15]. Because of its health benefits and a broad spectrum of traditional medicinal uses, this nutraceutical herb has been described in the literature as a miraculous herb [16], prophetic medicine [17], habbat al barakah [18], a holy herb of the Middle East [19], a mine for multi cure [20], a novel beauty care ingredient [21], a complementary drug [22], a universal healer [23], a marvel herb [24], a black seed of miracle [25], blessed seed [26], and a wonder herb [27].

Although all parts of the black seed herbaceous plant are of biological and economic importance, NS seeds and their oil (NSO) are most commonly consumed for their health benefits and to treat diseases in various indigenous systems of medicines [13]. NS seeds, owing to their peculiar aroma, fragrance, and nutritional value, are also used as food additives. The nutritional value of black seed is due to its rich content of vegetable proteins and amino acids, fats, fibers, carbohydrates, minerals, and vitamins. In traditional and indigenous medicine, different dosage forms of black seeds have been employed to manage multiple acute and chronic sicknesses, including respiratory diseases (asthma, bronchitis), pain and inflammation, skin allergy, hypertension, diabetes, liver disorders, rheumatism, mental disorder, malaria, loss of hunger, insomnia, snake bite, indigestion, and dysmenorrhea [28,29]. Numerous preclinical and clinical studies have explored the health-promoting potential of this traditional medicinal plant and provided evidence in favor of its antioxidant, antidiabetic, cardioprotective, neuroprotective, analgesic and anti-inflammatory, antimicrobial, anthelmintic, anti-oxytocic, anti-obesity, anti-cancer, anti-asthmatic, hepatoprotective, gastroprotective, anti-schistosomiasis, immunomodulatory, nephroprotective, and wound healing activities. Traditional uses, nutritional properties, and biological activities of NS have been thoroughly reviewed [13,15,28,29,30,31].

Phytochemical investigations of various plant parts of NS revealed it to contain various classes of secondary bioactive metabolites, such as terpenoids (thymoquinone, *p*-cymene, *trans*-anethole, carvacrol, limonene, dithymoquinone), alkaloids (nigellidine, nigellicimine, nigellicine, nigellicimine *N*-oxide, nigellamines), sterols (sitosterol, stigmasterol, tocopherol, tocotrienol), saponins (alpha-hederin, kalopanoxsaponin, nigella A-D), flavonoids (quercetin, kaempferol, rutin), phenolic acids (gallic acid, vanillic acid, *trans*-cinnamic acid) [13,28,29,32,33]. Black seeds on extraction with organic solvents or mechanical pressing yield approximately 34–39% of NS fixed oil (NSO) which primarily contains lipids (~98% to 99%) such as triacylglycerol, free fatty acids, sterols, and a small amount of volatile oil (1% to 2%) such as *p*-cymene and thymol [28,29]. Many preparations of NS seeds, seed oil, and extracts containing varying amounts of thymoquinone (TQ), either alone or in combination with other herbal products in capsule, powder, soft gels, and oil forms, are also available on the market. Black Cummin® of Bixabotanical is a proprietary Ayurvedic medicine for digestion to promote immunity and general health. Black seed oil containing 2% TQ extract, a brand of Health Thru Nutrition (HTN), is a dietary supplement available commercially as soft gels (500 mg). 

### 3.2. Important Biologically Active Chemical Constituents of NS 

**Figure 1:**
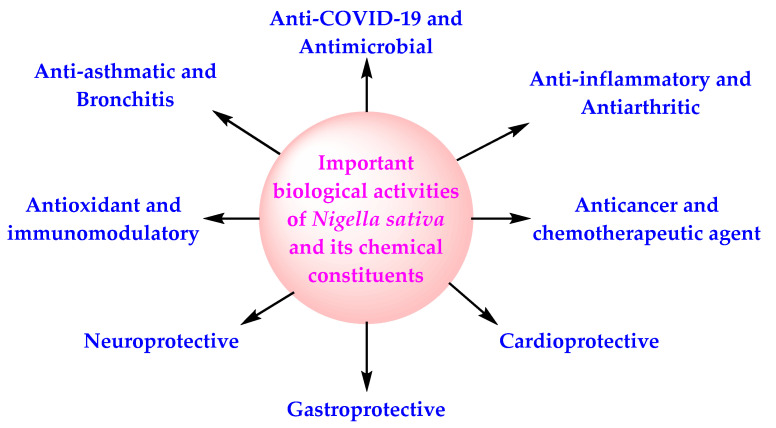
Therapeutic properties of NS and its chemical components.

**Figure 2:**
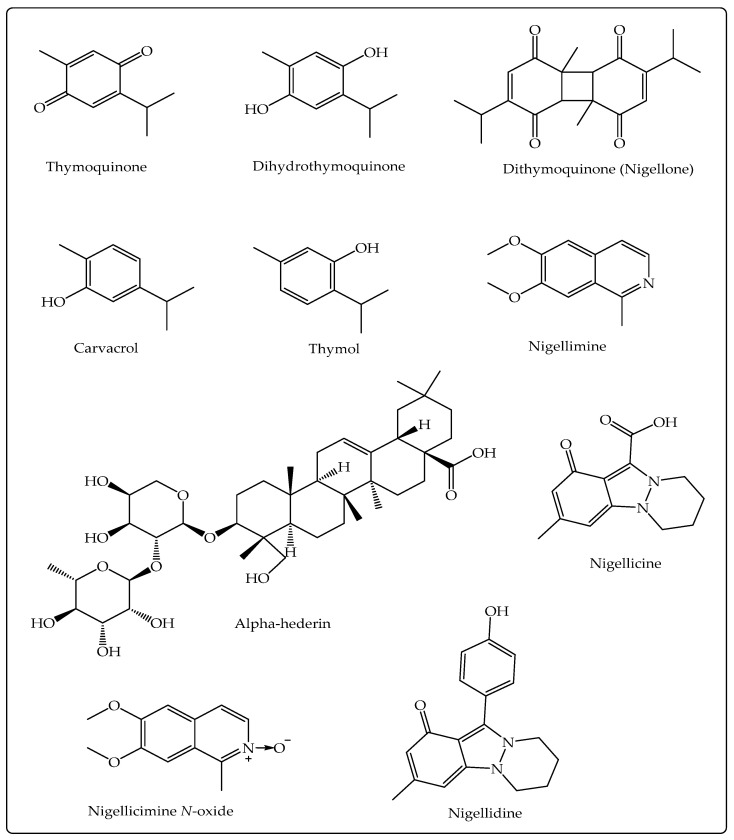
Chemical structure of the main bioactive constituents of *N. sativa* L. against COVID-19.

The pharmacological activities of NS, in general, are credited to the existence of volatile components, especially TQ, but other secondary metabolites (alkaloids, pentacyclic triterpenes, phytosterols, flavonoids), including those present in NSO, have also been shown to exhibit therapeutic activities [34,35,36,37,38] (Figure 1). The chemical structure of selected important bioactive components of NS is presented in Figure 2.

TQ (2-methyl-5-isopropyl-1, 4-benzoquinone; Figure 2) is a monoterpenoid and one of the key volatile components (30%–48%) of the NS volatile oil which exhibits an array of useful bioactivities [39]. It shows anti-inflammatory action by decreasing the appearance of pro-inflammatory cytokines (the COX-2, TNF-α, IL-6) and by upregulating the levels of IL-4 and IL-10, the anti-inflammatory cytokines [40]. Recently, Boskabady et al. demonstrated that TQ is effective in ameliorating lipopolysaccharide (LPS) generated lung inflammation in rats [41]. The immunomodulatory influences of TQ are mediated through activation of T cells, the appearance of CD62L and CD8+ T cells, and by helping in the formation of IFN-γ [42]. It is an effective chemotherapeutic agent against breast, bladder, cervical, colorectal, gastric, lung, prostate, pancreatic cancers, and leukemia in vitro and in vivo studies. It shows anticancer activity by producing apoptosis, autophagia, cell cycle arrest, controlling angiogenesis, and regulating signaling pathways [35,43,44]. Thymohydroquinone (THQ; Figure 2) exhibits stronger antioxidant activity and protects cell damage better than TQ [45]. Some other activities shown by TQ include neuroprotective, cardioprotective, antimicrobial, gastroprotective, antiarthritic, and anti-asthmatic activities [39]. Nigellone has been reported to inhibit trachea contraction along with improved mucociliary clearance and thus is useful in the treatment of bronchitis and asthma [46]. Alpha-Hederin, a water-soluble pentacyclic triterpene of NS, exhibits anticancer activity by inducing apoptosis, anti-inflammatory, and bronchodilatory activity by raising the levels of cAMP that leads to elevated β_2_-adrenergic responsiveness [47,48,49]. 

### 3.3. Anti-COVID-19 Chemical Constituents of NS

**Figure 3:**
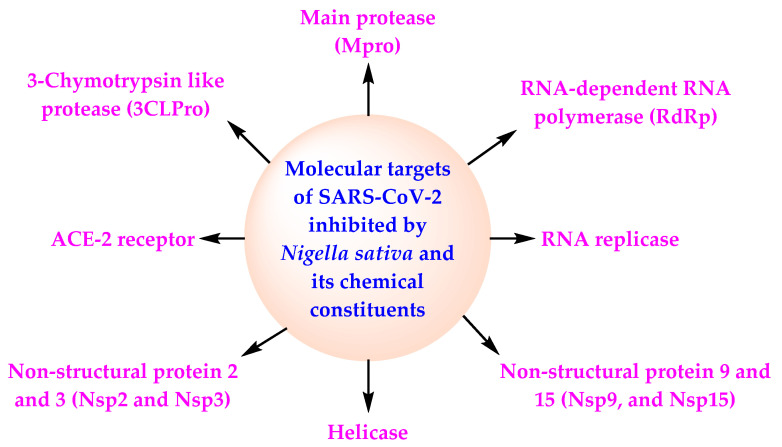
Mechanism of action of NS and its chemical constituents against SARS-CoV-2.

Several research publications reviewed the therapeutic activities of NS and TQ viz., antiviral, anti-inflammatory, immunomodulatory, antioxidant, and broncho-dilatory activities and suggested their possible role in combating COVID-19 [50,51,52,53,54]. A summary of the in silico, in vitro, and in vivo investigations done on bioactive phytoconstituents of NS since the break out of the COVID-19 pandemic is presented in Table 1. Data presented in Table 1 indicate that four in silico studies identified α-Hederin as the promising anti-COVID agent while DTQ in one in vitro and three in silico studies emerged as the lead anti-viral candidate. An in vivo study performed in experimental rats similarly exhibited the anti-SARS-CoV-2 efficacy of nigellidine [55]. Surprisingly, TQ was identified as a lead compound in only one in silico and one in vitro study. These phytochemicals have been shown to act at different molecular targets of SARS-CoV-2 (RdRp, 3CL^pro^, Nsp15, Nsp2, Nsp3, M^pro^, and ACE2) (Figure 3).

### 3.4. Anti-COVID-19 Clinical Trials on NS and Its Chemical Constituents

A search for the clinical studies related to NS was conducted on the clinical trial database [67] utilizing the terms “*N. sativa* or its synonyms” and the name of its different important chemical constituents (TQ, THQ, DTQ or nigellone, nigellimine, nigellicine, nigellidine, nigellicimine, and nigellicimine *N*-oxide) on 20 February 2022. This search provided eight clinical studies belonging to NS and TQ to treat/prevent COVID-19. Two CTs have been conducted in Saudi Arabia, two in Pakistan, two in Tunisia, and one each in the United States, and Egypt. Two CTs were related to the prevention of COVID-19, whereas six CTs involved treatment of COVID-19 utilizing NS. Most of the CTs have been conducted employing the capsule (500 mg) containing an effective amount of NS and/or NSO. The CTs have also been done using a combination of NS and honey or Omega 3. The data of this search are summarized in Table 2.

## 4. Patent Summary

A patent search was carried out on 20 February 2022, using patent databases as mentioned in the literature [68,69,70,71]. The summary of the finalized patents/patent application is provided in Table 3.

## 5. Conclusions

The supplementation of the NS possesses a variety of advantageous effects on human well-being. Several compositions comprising NS and NSO have been tested clinically and have also been claimed in patent applications to prevent/treat COVID-19. The clinical studies and patent filings support the therapeutic efficacy of NS against COVID-19 owing to its immunomodulatory, antioxidant, antiviral, and anti-inflammatory effects. NS can prevent and treat COVID-19 patients with a faster recovery rate. Many compositions of NS in combination with many medicinal herbs and medicines are still unexplored. However, further clinical trials are recommended to establish the anti-COVID-19 potential of the unexplored NS compositions.

## 6. Discussion and Perspective

NS is one of the Quranic and prophetic medicinal plants with beneficial effects on human health [87]. The literature and clinical studies have demonstrated its efficacy in preventing and treating COVID-19. The summary of eight CTs on NS seeds and NSO is mentioned in Table 1. The data of six CTs have not been published. However, the data of two CTs (NCT04401202 and NCT04347382) involving the use of NS against COVID-19 have been published [52,88].

One of the published clinical studies (NCT04401202) concluded that NSO supplementation provides faster recovery of 62% of the mild COVID-19 patients on day 14 of the treatment. The normal revival time was also briefer than the control group. This study suggested that the reduction in the COVID-19 symptoms (anosmia, chills, runny nose, and loss of appetite) might be owing to the anti-inflammatory properties of NS [52]. The infection of SARS-CoV-2 causes the cytokine storm because of the liberation of leukocytes and pro-inflammatory cytokines [89,90]. The discussion part of the published CTs [52] stated that NS demonstrates immunostimulant and anti-inflammatory effects by restricting the release of pro-inflammatory cytokines [54]. In addition, NS also displays antioxidant and antiviral effects [54]. Accordingly, drugs like NS can stop the cytokine storm and are useful to prevent/treat COVID-19.

The published results of another CT (NCT04347382) affirmed that the combination of honey and NSO improved the COVID-19 symptoms, viral clearance, and mortality among COVID-19 patients. This report also advocated the use of the combination of honey and NS along with other treatments of COVID-19 [88]. According to this study, the combination of honey and NS provided its anti-COVID-19 activity due to their antioxidative/antiviral/immunostimulant chemical constituents (phenolic compounds, flavonoids, and zinc) that attack the multiple sites (lowering the expression of ACE-2 receptor, RdRp, Mpro protease, etc.,) of SARS-CoV-2. 

A CT (IRCT20180712040449N2) was conducted in Iran utilizing a combination of 500 mg of capsule-1 containing hydroethanolic extract of *Rheum palmatum* (root), *Glycyrrhiza glabra* (rhizome), and *Punica granatum* (fruit peel) (0.5:1:1), capsule-2 containing 500 mg of NS seed powder, and a polyherbal water decoction prepared from a herbal mixture (1 g each herb in the same sachet) comprising *Matricaria chamomilla* (flower), *Zataria multiflora* (aerial part), *Glycyrrhiza glabra* (root), *Ficus carica* (fruit), *Urtica dioica* (leaf), *Althaea officinalis* (flower), and *Nepeta bracteate* (flower) [91]. Capsule-1 and capsule-2 were administered to patients two times a day and the polyherbal decoction two times a day along with the standard care. This treatment significantly reduced the hospital dyspnea, accelerated recovery time, and lowered the COVID-19 symptoms. This study implicitly indicates that the chemical constituents of NS (TQ, hederagenin, THQ, nigelledine, and α-hederin) are anti-COVID-19 compounds.

Many in silico, in vitro, and in vivo studies have demonstrated potential anti-COVID-19 activity of the main chemical constituents of NS due to their diverse mechanisms of action against SARS-CoV-2 (Table 1) (Figure 3). TQ is one of the main chemical constituents of NSO possessing antioxidant, anti-inflammatory, and antiviral activities [31,92]. It is disclosed that TQ may bind the hydrophobic component of SARS-CoV-2 because of its lipophilicity. This causes activation of the virus. TQ also reduces the generation of cytokine storm in COVID-19 patients because of its anti-inflammatory potential [93]. TQ has also demonstrated SARS-CoV-2 inhibitory activity and can be an efficacious adjuvant for the therapy of COVID-19 [94].

Many findings have also established the potential of NS seeds and TQ in reducing thrombus formation [95]. It is a well-known fact that thrombus formation causes multiple organ collapse and fatality among COVID-19 patients. Therefore, NS may be used as a therapeutic formulation, including its nano-formulations [96] to treat COVID-19, and may also be used as a supportive therapy with anti-COVID-19 medicines [4,6,97,98].

NS and its oil possess antiviral activity, cardioprotective activity, gastroprotective activity, neuroprotective activity, anticancer activity, antidiabetic activity, antioxidant activity, anti-obesity activity, immunomodulatory activity, antihistaminic activity, and anti-inflammatory activity [99,100] (Figure 1). NS and NSO inhibit the activity of SARS-CoV-2 through the diverse mechanism of action (Figure 3). All these facts, in combination with the data of the CTs based on NS, indicate the potential of NS for the management of COVID-19 in normal COVID-19 patients as well as among the high-risk population.

A patent application is filed for a novel and inventive research of industrial application [101]. Our search revealed several patent applications connected to the practice of different compositions of NS to prevent/treat COVID-19 (Table 2). Most of these patents were published in 2021. Like non-patent literature, the patent literature also cites NS as an immunomodulator (activates natural killer cells, interferon, and dendritic cells), suppresses virus multiplication by its chemical constituents like TQ, protects lungs from pathology by blocking the activities of several inflammatory mediators that perform a major function in the COVID-19 pathology, several chemical constituents of NS, including TQ, nigellidine, and alpha-heridin have displayed anti-COVID-19 activity, the extracts of NS showed BCG like activity, and many NS compositions are in clinical trial against COVID-19 [73,75,77].

NS also has antihistaminic, antioxidant, antiallergic, and anti-asthmatic effects [74]. NS is also a source of zinc [77], which has also demonstrated activity against SARS-CoV-2 [102]. Many publications have also stated the usefulness of NS against chronic diseases like cancer, cardiovascular diseases, and diabetes, as mentioned above [78]. It is acceptably recognized that immunocompromised patients with little levels of natural killer cells, interferon, and dendritic cells are vulnerable to SARS-CoV-2 infection. COVID-19 patients suffer from cytokine storm owing to increased activity of the pro-inflammatory mediators in the body, and the malfunction of the lung among COVID-19 patients lead to severe consequences [73,75,77]. All these facts indicate that NS and its various compositions have all the attributes to be used as a promising remedy for the management of COVID-19 among high-risk individuals as well as to treat normal COVID-19 patients as a monotherapy or a combination therapy.

## Figures and Tables

**Table 1:** Summary of several in silico, in vitro, and in vivo investigations carried out on NS chemical constituents against COVID-19.

NS Constituent Identified as Anti-COVID-19	Type of Study	Finding of the Study
Caryophyllene oxide, β-bisabolene	In silico	Molecular docking studies (PDB IDs: 6YHU, 6W4B, 6VXS, 6LU7, 7BTF 6LZG) revealed caryophyllene oxide to possess the highest binding affinity towards 3CLpro, NSP3, NSP9, and RdRp molecular targets in COVID-19. ACE-2 binding affinity of β-bisabolene and remdesivir was almost similar [56]
Dithymoquinone (DTQ)	In silico	The binding affinity (PDB ID: 6VW1) of DTQ on SARS-CoV-2-ACE-2 was better than chloroquine. It was found to be stable at the docked site in molecular dynamics simulation studies [57]
α-Hederin	In silico	α-Hederin was found to be a better inhibitor of RdRp (PDB ID: 6M71) than DTQ, nigellicine, and nigellidine [58]
DTQ	In silico	DTQ was found to be active against 3CLpro (PDB ID: 6LU7) and Nsp15 (PDB ID: 6VWW) targets [59]
α -Hederin, rutin, and nigellamine A2	In silico	α-Hederin, rutin, and nigellamine A2 were identified as potential inhibitors of SARS-CoV-2 proteins (PDB IDs: 6W9C, 6Y2E, 6M71, 6ZSL, 6W4B, 6VWW, 6M17, and 6VYO) related to RdRp, protease, and helicase [60]
Nigellidine	In silico and in vivo	Nigellidine showed a good affinity toward COVID-19 Nsp2 and IL1R proteins (PDB IDs: 6LU7, 6VSB, 1ITB, and 1P9M). Nigellidine in vivo study in rats showed antioxidant, hepato-protective, and anti-inflammatory activities [61]
α-Hederin	In silico	NS chemical constituents such as α-hederin, stigmasterol glucoside, nigellidine-4-O-sulfite, nigellidine, sterol-3-β-D-glucoside, DTQ, β-sitosterol were identified as potential inhibitors of main protease (M^pro^) (PDB IDs: 6LU7). Nigllimine, nigellimine *N*-oxide, carvacrol, TQ, THQ, thymol, anthole, etc., showed weaker binding affinity than remdesivir, lopinavir, and nelfinavir. α-Hederin was identified as the most promising anti-COVID agent [62]
α-Hederin, THQ, and TQ	In silico	In molecular docking studies, α-hederin, THQ, and TQ were found to be efficiently binding to ACE-2 (PDB ID: 1R4L) of SARS-CoV-2 [63]
Nigellone	In silico	Nigellone (DTQ) upon molecular docking studies with four COVID-19 protein targets (spike glycoprotein, 3CL^pro^/M^pro^ (PDB ID: 6LU7), human ACE-2) was observed to bind more strongly than carvacrol, nigellicine, nigellidine, TQ, THQ, and thymol. Its binding affinity on other viral proteins (PDB IDs: 6LU7, 6VSB, and 6VX) was better than remdesivir and hydroxychloroquine [64]. This study also advocated further in vitro experiments to establish Nigellone as an anti-COVID-19 lead compound
Nigellidine	In silico	Nigellidine was exposed to prevent SARS-CoV-2 NSP3 replication/transcription. It also blocked the pro-inflammatory cytokines TNF R1 and TNF R2 and Fas-induced apoptotic death [55]
DTQ and THQ	In vitro	Cytotoxicity of DTQ and THQ was tested in VERO-E6 cells by MTT assay. HTQ presented anti-SARS-CoV-2 action at non-cytotoxic nanomolar concentration (IC_50_ = 23.15 ng/mL) while DTQ showed an IC_50_ of 275.2 ng/mL [65]
TQ	In Silico and in vitro	TQ is bound strongly to ACE-2 of SARS-CoV-2 (PDB ID: 6VW1). In vitro results showed it to inhibit SARS-CoV-2 pseudo particles infecting HEK293-ACE2 cells with IC_50_ of 4.999 μM and CC_50_ of 35.100 μM; SI = 7.02) [66]

**Table 2:** Interventional clinical studies on NS against COVID-19/SARS-CoV-2.

Summary of the Title (Intervention)	Primary Purpose (Phase; Number of Enrollments; Status; Results)	NCT Number (Allocation; Intervention Model; Completion Date)	Sponsor (Location of the Clinical Trial)	Primary Outcomes/Conclusion
NS in COVID-19 (Oral soft gel capsule containing 500 mg NSO two times a day for 10 days)	Treatment (2; 183; Completed; Available)	NCT04401202 (Randomized; Parallel Assignment; 31 December 2020)	King Abdulaziz University (Saudi Arabi)	The dietary supplement helped the faster recovery of COVID-19 patients
Safety and efficacy of NSO against COVID-19 (Six 500 mg capsules of NSO per day for 14 days)	Treatment (2; 60; Recruiting; Not available)	NCT04914377 (Randomized; Parallel Assignment; November 2021)	Novatek Pharmaceuticals (United States)	The reduction in the COVID-19 signs and symptoms
Effectiveness of NSO to treat COVID-19 (One capsule of NS every 2 h for the first 3 days followed by one capsule three times a day for 12 days. The dose of NSO is not mentioned)	Treatment (1; 500; Completed; Not available)	NCT04914767 (Randomized; Parallel Assignment; 31 December 2021)	Sahloul University Hospital (Tunisia)	Rate of death, readmission, and oxygen supplementation among high-risk COVID-19 patients
Honey and NS seeds for COVID-19 treatment (Honey 1 g/kg daily + NS seed capsule, 80 mg/kg daily for 14 days)	Treatment (3; 313; Completed; Not available)	NCT04347382 (Randomized; Parallel Assignment; 30 August 2020)	Sohaib Ashraf and Sheikh Zayed Federal Postgraduate Medical Institute (Pakistan)	Days needed to obtain a negative COVID-19 PCR of a COVID-19 positive patient
Efficacy of NS versus vitamin D3 against COVID-19 (NS capsule, 900 mg two times a day for 14 days)	Treatment (Not Applicable; 100; Recruiting; Not available)	NCT04981743 (Randomized; Parallel Assignment; 30 December 2021)	Ain Shams University (Egypt)	The safety and efficacy of NS versus vitamin D3 against COVID-19 will be evaluated and recorded utilizing COVID-19 signs and symptoms (fever, runny nose, fatigue, cough, sore throat, and headache)
Impact of the composition of NSO and Omega 3 on the immunity of COVID-19 patient (1g Omega 3 and 1g NSO containing 3% TQ for 14 days)	Treatment (2 & 3; Recruiting; Not available)	NCT04553705 (Randomized; Sequential Assignment; 4 December 2020)	Beni-Suef University, Maternity and Children Hospital (Makkah), and University of Arizona (Saudi Arabia)	The recovery rate from COVID-19 positive to COVID-19 negative
NS for the prevention of influenza syndrome (One capsule of NS per day for 21 days followed by weekly follow-up for COVID-19 checking. The dose is not mentioned)	Prevention (Not applicable; 500; Completed; Not available)	NCT04989101 (Randomized; Parallel Assignment; 31 August 2021)	Sahloul University Hospital (Tunisia)	SARS-CoV-19 infection
Honey and NS seeds for COVID-19 prophylaxis (Honey 0.5 g/kg daily + NS seeds 40 mg/kg daily for 14 days)	Prevention (2 & 3; 1000; Recruiting; Not available)	NCT04767087 (Randomized; Parallel Assignment; 15 April 2022)	Sohaib Ashraf and Sheikh Zayed Federal Postgraduate Medical Institute (Pakistan)	The combination of honey and NS improved the symptoms, viral clearance, and mortality among COVID-19 patients

**Table 3:** Summary of the patent literature.

Patent/Patent Application Number (Applicant/Assignee; Publication Date; Priority Country)	Status (Family Members; International Patent Classification)	Summary of the Claimed Invention
US11229674B1 (Therapeutic Solutions International; 25 January 2022; United States)	Patented case (None; A61K36/31, A61K36/45, A61K36/71, A61K36/82, A61P29/00)	A quadramune composition comprising 100–200 ug of green tea extract (epigallocatechin-3-gallate), 100–200 ug of NS extract (TQ), 100–200 ug of broccoli extract (sulforaphane), and 50-100 ug of blueberry extract (pterostilbene) to treat COVID-19 patient. This composition is said to possess anti-inflammatory activity and improves immunity by inhibiting the expression of indoleamine 2,3-dioxygenase. However, no clinical or in vitro analysis data have been provided in support of the claimed method of treatment [72].
US20210338763A1 (Therapeutic Solutions International; 4 November 2021; United States)	Under examination (None; A61K31/09, A61K31/122, A61K31/26, A61K31/353, A61K36/31, A61K36/45, A61K36/71, A61K36/82)	It claims a nutraceutical composition similar to US11229674B1 [72] comprising NS, green tea, blueberry, and broccoli for treating or preventing complications linked with the infection of SARS-CoV-2. The composition is claimed to reduce the expression of inflammatory markers in the human body. No example has been provided in the specification to support the claimed invention, but inventors tried to justify their claims based on prior studies [73].
WO2022009236A1 (Mozhdeh Haddadi and Mahdyar Taghdisi Hadi Pour; 13 January 2022; Iran)	No national phase entry (A61K36/00)	It claims four types of compositions of NS (capsule/tablet) for the treatment of COVID-19 (10 days course) containing different dry and powdered herbs. First composition for patients < 3 years comprised of NS and *Terminalia chebula* (TC). Second composition for patients of 3–7 years comprised of NS, Apple seed, and TC. Third composition for patients 7–15 years comprised of NS, *Peganum harmala* (PH), and TC. Fourth composition for patients >15 years NS, PH, Apple seed, and TC. This document does not deliver any experimental proof (in vitro, in vivo, or clinical) for the claimed treatment [74].
US20220000958A1 (Covimmune Pharma; 6 January 2022; United States)	Under examination (A61K36/71, A61P31/14)	A biologically active immunostimulant extract obtained by the extraction of NS plant, NS seed, or its oil with aqueous ethanoic acid (vinegar) for the treatment of COVID-19. The patent application provides a pictorial mechanism of action of the NS extract to treat COVID-19. However, no anti-COVID-19 activity data (in vitro, in vivo, and clinical) of the extract have been exemplified [75].
TR2020004046A2 (Alravvi, Omar, Turk; 21 April 2020; Turkey)	Granted patent (Not available online)	An antiviral herbal composition comprising NSO (60–80%), olive oil (10–20%), and clove oil (1–5%) for treating COVID-19. The complete document was not available for analyzing the examples [76].
WO2021205196A1 (Muhammad Taliah; 14 October 2021; International Bureau of The World Intellectual Property Organization)	No national phase entry (WO2021205196A4; A61K36/19, A61K36/38, A61K36/48, A61K36/70, A61P31/14)	A composition comprising NS seed (anti-SARS-CoV-2) and *Saussurea lappa* root (anti-SARS-CoV-2) for treating/preventing COVID-19. The optional components of the composition include, *Astragalus membranaceus* root (immunomodulator/reduces viral load), *Paeonia lactiflora* root (anti-inflammatory/immunomodulator), *Radix bupleuri* root (anti-inflammatory), *Nelumbo nucifera* seed (antioxidant), *Angelica archangelica* root (antioxidant), *Citrus sinensis* peel (antioxidant), *Rosa canina* fruit (antioxidant), *Vaccinium angustifolium* fruit (antioxidant), *Polygonum cuspidatum* root (antioxidant/antiviral), *Ocimum sanctum* leaf (antiviral), *Andrographis paniculate* aerial parts (antiviral), *Artemisia vulgaris* leaf (autophagy inducer), *Zingiber officinale* root (autophagy inducer), *Glycyrrhiza glabra* root (antimutagenic), and *Panax ginseng* root (reduces lethargy and fatigue associated with COVID-19). This patent application provides a protocol for the clinical study of the claimed composition but is silent about its data [77].
US2022023237A1 (Therapeutic Solutions International; 27 January 2022; United States)	Under examination (None; A23L33/105, A61K31/155, A61K36/31, A61K36/45, A61K36/82)	A synergistic composition to treat/prevent COVID-19 comprising metformin, Green Tea extract (epigallocatechin-3-gallate), blueberry extract (pterostilebene), NS extract (TQ), and broccoli extract (sulforaphane). The in vivo activity data demonstrated that the claimed composition enhanced type 2 monocytes, IL-10 (anti-inflammatory protein), and HGF-1 (regenerative protein). It also reduced lung injury, and IL-17 (inflammatory protein). However, no specific data have been provided against SARS-CoV-2 or COVID-19 treatment [78].
US2022031793A1 (Therapeutic Solutions International; 3 February 2022; United States)	Under examination (None; A61K31/09, A61K31/122, A61K31/26, A61K31/353, A61K36/31, A61K36/45, A61K36/71, A61K36/82)	A method of protecting against neurological damage due to COVID-19 utilizing a composition comprising green tea extract (epigallocatechin-3-gallate), blueberry extract (pterostilbene), NS extract (TQ), and broccoli extract (sulforaphane) [79].
US2022040248A1 (Therapeutic Solutions International; 10 February 2022; United States)	Under examination (None; A61K31/09, A61K31/122, A61K31/26, A61K31/353, A61K36/31, A61K36/45, A61K36/71, A61K36/82, A61P25/28)	A method of treating inflammation associated with neurological damage due to COVID-19 utilizing a composition comprising green tea extract (epigallocatechin-3-gallate), blueberry extract (pterostilbene), NS extract (TQ), and broccoli extract (sulforaphane) [80].
WO2021186453A1 (Alkalay Rachel; 23 September 2021; United States)	No national phase entry (WO2021186454A1, WO2021186455A1, WO2021186456A1; A61K31/05, A61K36/25, A61K36/258, A61K36/324, A61K36/424, A61K36/53, A61K36/537, A61P11/00, A61P31/14)	A method of preventing or treating SARS-CoV-2 infection using a composition containing oregano oil (1), thyme oil (2), NSO (3), sumac oil (4), sesame oil (5), olibanum oil (6). Different combinations of these oils were made as combination A (1+2+3), combination B (1+2+3+4), combination C (1+2+3+4+5), and combination D (1+2+3+4+5+6). The in vitro analysis of these combinations demonstrated that these combinations digested the S-1 and S-2 subunits of the spike protein of SARS-CoV-2 and attenuated it. The clinical trial in a patient showed that the combination-A relieved sore throat and cough in 24 and 40 h, respectively [81].
WO2021186454A1 (Alkalay Rachel; 23 September 2021; United States)	No national phase entry (WO2021186453A1, WO2021186455A1, WO2021186456A1; A61K31/05, A61K36/25, A61K36/258, A61K36/324, A61K36/424, A61K36/53, A61K36/537, A61P11/00, A61P31/14)	This is a family member of WO2021186453A1 [81] with similar data. It claims a method of lowering the infectivity of a non-malignant respiratory disease virus using a combination of NS along with other herbs as mentioned in WO2021186453A1 [81,82].
WO2021186455A1 (Alkalay Rachel; 23 September 2021; United State)	No national phase entry (WO2021186453A1, WO2021186454A1, WO2021186456A1; A61K31/05, A61K36/25, A61K36/258, A61K36/324, A61K36/424, A61K36/53, A61K36/537, A61P31/12, C07K14/47)	This is a family member of WO2021186453A1 [81] with similar data. It claims an anti-inflammatory composition of NS along with other herbs as mentioned in WO2021186453A1 [81,83].
WO2021186456A1 (Alkalay Rachel; 23 September 2021; United States)	No national phase entry (WO2021186453A1, WO2021186454A1, WO2021186455A1; A61K31/045, A61K31/05, A61K31/121, A61K31/198, A61K31/352, A61K36/258, A61K36/424, A61K38/48, A61P35/00)	This is a family member of WO2021186453A1 [81] with similar data. It claims the antiviral composition of NS along with other herbs as mentioned in WO2021186453A1 [81]. However, it is silent about the antiviral activity against SARS-CoV-2 [81,84].
WO2021160982A1 (Nasaleze Patents Limited; 19 August 2021; United Kingdom)	No national phase entry (None; A61K31/685, A61K36/8962, A61K47/38, A61K47/46, A61K9/00, A61K9/14, A61P31/12)	A homogenized powdered composition consisting of hydroxypropyl methylcellulose particles, at least one signaling agent (menthol, strawberry, mint, spearmint, peppermint, eucalyptus, lavender, and citrus), and optionally one or more biologically active agents like NS. This document does not furnish any rationale for using NS in the description part [85].
WO2021216749A1 (Hoag George Edward and Salerno John; 28 October 2021; United States)	No national phase entry (None; A61K31/015, A61K31/045, A61K31/12, A61K31/35, A61K9/00, A61P31/04, A61P31/12)	A liquid pharmaceutical composition for inhalation to prevent/treat infectious diseases (COVID-19) containing a plant extract comprising one or more Transient Receptor Potential Cation Channel, Subfamily A, member 1 (TRPA1) antagonist (1,8-cineole), one or more plant extract antibacterial compound (b-caryophyllene), one or more plant extract antiviral compounds (TQ), and one or more plant extract antioxidants (berberine). This patent application does not exemplify the anti-SARS-CoV-2 activity data of the claimed composition [86].

## Supplementary Materials

The following supporting information can be downloaded at: https://www.mdpi.com/article/10.3390/molecules27092750/s1, Table S1: Patent searching results.
