# Supplementary material for: Nigella sativa L. and COVID-19: A Glance at The Anti-COVID-19 Chemical Constituents, Clinical Trials, Inventions, and Patent Literature
Source: Molecules. 2022 Apr 25;27(9):2750. doi: 10.3390/molecules27092750 (PMC9105261; doi:10.3390/molecules27092750)
Supplement: Supplementary file 1 [file molecules-27-02750-s001.zip › molecules-1649433-supplementary.pdf]

Table S1: Patent searching results

Patent database	Keyword combination	Number of hits
Sci-Finder	Black cumin + Covid-19	1
	Black seed + Covid-19	2
	Kalonji + Covid-19	1
	Nigella + SARS-CoV-2	7
	Nigella + Covid-19	6
	Nigellicine + Covid-19	0
	Nigellicine + SARS-CoV-2	0
	Nigellimine + Covid-19	0
	Nigellimine + SARS-CoV-2	0
	Sativa + Covid-19	20
	Sativa + SARS-CoV-2	18
	Thymoquinone + Covid-19	4
	Thymoquinone + SARS-CoV-2	3
Espacenet	Black cumin + Covid-19	1
	Black seed + Covid-19	5
	Kalonji + Covid-19	0
	Nigella + SARS-CoV-2	2
	Nigella + Covid-19	7
	Nigellicine + Covid-19	0
	Nigellicine + SARS-CoV-2	0
	Nigellimine + Covid-19	0
	Nigellimine + SARS-CoV-2	0
	Sativa + Covid-19	17
	Sativa + SARS-CoV-2	8
	Thymoquinone + Covid-19	6

	Thymoquinone + SARS-CoV-2	6
**Patentscope**	Black cumin + Covid-19	1
	Black seed + Covid-19	6
	Kalonji + Covid-19	0
	Nigella + Covid-19	5
	Nigellicine + Covid-19	0
	Nigellicine + SARS-CoV-2	0
	Nigellimine + Covid-19	0
	Nigellimine + SARS-CoV-2	0
	Sativa + Covid-19	15
	Sativa + SARS-CoV-2	10
	Thymoquinone + Covid-19	3
	Thymoquinone + SARS-CoV-2	8
**USPTO**	Black cumin + Covid-19	0
	Black seed + Covid-19	7
	Kalonji + Covid-19	0
	Nigella + SARS-CoV-2	2
	Nigella + Covid-19	2
	Nigellicine + Covid-19	0
	Nigellicine + SARS-CoV-2	0
	Nigellimine + Covid-19	0
	Nigellimine + SARS-CoV-2	0
	Sativa + Covid-19	4
	Sativa + SARS-CoV-2	3
	Thymoquinone + Covid-19	2
	Thymoquinone + SARS-CoV-2	2
